# The preoperative hemoglobin, albumin, lymphocyte and platelet (HALP) score is a useful predictor in patients with resectable esophageal squamous cell carcinoma

**DOI:** 10.17305/bjbms.2021.5666

**Published:** 2021-12

**Authors:** Ji-Feng Feng, Liang Wang, Xun Yang

**Affiliations:** Department of Thoracic Oncological Surgery, Institute of Cancer Research and Basic Medical Sciences of Chinese Academy of Sciences, Cancer Hospital of University of Chinese Academy of Sciences, Zhejiang Cancer Hospital, Hangzhou, China

**Keywords:** Esophageal squamous cell carcinoma, neutrophil to lymphocyte ratio, platelet to lymphocyte ratio, hemoglobin, albumin, cancer-specific survival

## Abstract

The hemoglobin, albumin, lymphocyte and platelet (HALP) score has been confirmed as a prognostic factor in several types of cancers. The current study aimed to assess the prognostic value of preoperative HALP score, an inflammatory and nutritional based score, in predicting cancer-specific survival (CSS) in resectable patients undergoing curative resection for esophageal squamous cell carcinoma (ESCC). The clinical data of 355 consecutive patients with ESCC who underwent curative resection were retrospectively conducted and analyzed. The receiver operating characteristic (ROC) curve was used to determine the optimal cut-off value for preoperative HALP. The areas under the curve (AUC) for preoperative HALP and other variables were calculated and compared. Cox regression analyses and Kaplan–Meier methods were used to identify the factors associated with CSS. According to the ROC curve, the optimal cut-off value for preoperative HALP was 31.8. The 5-year CSS for preoperative HALP low (≤31.8) and high (>31.8) was 15.1% and 47.5%, respectively (p < 0.001). Preoperative HALP had reliable abilities to predict CSS in resectable ESCC patients in any stage or gender, according to the subgroup analysis based on the patients’ cancer stage and gender. Multivariate analyses confirmed that preoperative HALP was an independent prognostic score regarding CSS in patients with resectable ESCC (p < 0.001). This study confirmed that the postoperative HALP score could be regarded as a potential independent prognostic factor for CSS in patients with resectable ESCC.

## INTRODUCTION

Esophageal cancer (EC) is one of the most common malignancies in the world [1]. The highest-risk area of so-called “Asian EC Belt” includes Kazakhstan, Iran, Turkey and northern and central China, with an estimated incidence more than 100/100,000 [2]. In China, EC is the fourth most common cause of cancer death [3]. Although the incidence of EC has declined in China over the past few decades, it remains a significant public health burden. China accounted for nearly half of the global new cases of EC in 2012, with an estimated 0.287 (21.17/100,000) million new cases and 0.211 (15.58/100,000) million death cases [4]. Approximately 2.338 million cancer deaths (1.48 million for men and 0.858 million for women) were reported in 2015 in China, of which 0.188 million died from EC (0.137 million for men and 0.051 million for women) with a crude mortality rate of 13.68/100,000 [3]. There are two main histologic types in EC: adenocarcinoma (AC) and squamous cell carcinoma (SCC), of which esophageal SCC (ESCC) accounts for more than 90% in China [5]. Although the treatment is improved due to the advance in medical technology in recent years, the prognosis in patients with EC is still poor, mainly due to the fact that most patients are diagnosed at the time of advanced stage [6,7]. According to the main characteristics of ESCC in China, therefore, it is urgent to explore more and more prognostic factors.

It is well known that nutritional and inflammatory status is associated with cancer prognosis. In recent years, therefore, a variety of inflammation and/or nutrition-related predictors, including platelet to lymphocyte ratio (PLR), prognostic nutritional index (PNI), and neutrophil to lymphocyte ratio (NLR) have been used in various cancers, including ESCC [8-12]. Hemoglobin, albumin, lymphocyte and platelet (HALP) score, consisting of four laboratory markers in both nutritional and inflammatory status, including HALP is recently proposed to predict the prognosis in patients with several types of cancers [13-16]. However, the prognostic value of HALP score in patients with EC remains unclear. To the best of our knowledge, only one study, including 39 patients, has concluded that HALP could predict the chemoradiotherapy response based on platinum in male patients with ESCC [17]. The previous published study of ESCC focused on inoperable patients with small sample (39 patients). Therefore, the current study aimed to determine the prognostic role of postoperative HALP score in predicting cancer-specific survival (CSS) in patients with resectable ESCC. Furthermore, the current study also aimed to analyze the comparisons of prediction accuracy between postoperative HALP and its components of four laboratory markers (albumin, lymphocyte, hemoglobin, and platelet) in both nutritional and inflammatory status as well as other prognostic scores such as NLR, PLR, and PNI.

## MATERIALS AND METHODS

### Patient selection

A retrospective study was conducted and analyzed, including 355 consecutive ESCC cases with radical resection from June 2011 to March 2013. The following inclusion criteria were included in this study: (a) ESCC was pathologically confirmed, (b) treatment with radical resection (R0 resection), (c) patients without distant metastases, (d) patients without any neoadjuvant treatments before radical resection, and (e) detailed clinical data and follow-up records including postoperative laboratory results obtained within 1 week before surgery. Patients were excluded according to the following criteria: (a) Patients without R0 resection (R1 or R2 resection), (b) patients with distant metastases, (c) patients treated with neoadjuvant treatments before surgery, (d) patients with other pathological types, (e) ESCC combined with other malignant tumors, (f) severe complications or deaths within 30 days after surgery, (g) patients with infection or autoimmune disease, (H) patients with incomplete clinical data or postoperative laboratory results, and (I) patients without follow-up data.

### Treatment and follow-up

To date, esophagectomy with lymphadenectomy was the main surgical technique for EC. The procedures of McKeown and Ivor Lewis were two main surgical procedures for thoracic esophagectomy [18,19]. The 7^th^ AJCC/UICC TNM staging system was performed in the current study [20]. Patients with a high depth of tumor invasion and/or lymphatic metastasis were treated with adjuvant treatments, including radiotherapy (45-50.4 Gy) and/or chemotherapy (cisplatin and 5-fluorouracil-based regimes) after resection, but adjuvant treatments were not mandatory. Patients were followed-up regularly (every 3 months in the first 2 years, every 6 months in the next 3 years, and once a year after 5 years) after radical resection. The followed-up included clinical examinations, tests of tumor markers, and computerized tomography (CT) scan of the chest and abdomen (enhanced CT if necessary). Endoscopic biopsy was performed when clinical or radiographic evidence revealed a local recurrence. Positron emission tomography-CT was conducted if necessary. The follow-up results were obtained from our medical records. The last follow-up was completed in June 2019.

### Data collection

The laboratory results, according to the medical records, were obtained within 1 week before surgery, such as lymphocyte, neutrophil, hemoglobin, platelet, and albumin. The levels of lymphocyte, neutrophil, hemoglobin, and platelet counts were obtained from an automated blood cell counter (Sysmex XE-2100, Kobe, Japan). The albumin level was measured by an automated biochemical analyzer (Hitachi 917, Munich, Germany). The PLR and NLR were defined as the ratios of platelet to lymphocyte counts and neutrophil to lymphocyte counts, respectively. The PNI was calculated as albumin level (g/L) + 5 × lymphocyte (10^9^/L). The definition of HALP was determined by hemoglobin (g/L) × albumin (g/L) × lymphocyte (/L)/platelet (/L) [13-17].

### Ethical statement

The current study was conducted in accordance with the Helsinki Declaration and approved by the ethics committee of Zhejiang Cancer Hospital (No.2020-031).

### Statistical analysis

MedCalc 17.6 (MedCalc Software, Ostend, Belgium) was used to conduct all statistical analyses. The optimal cut-off points for postoperative HALP and other prognostic scores (NLR, PLR, and PNI) were selected by receiver operating characteristic (ROC) curves according to the CSS status (alive and death). The areas under the curve (AUC) for HALP and its components of HALP, as well as NLR, PLR, and PNI, were calculated and compared in both continuous and categorical statuses. The Fisher’s exact tests or Chi-squared tests for categorical variables and t-tests for continuous variables were used to evaluate the correlations grouped by HALP. The associations between CSS and prognostic factors with univariate and multivariate were conducted and analyzed by the Cox regression analyses and Kaplan–Meier methods. Hazard ratios (HRs) with 95% confidence intervals (CIs) were also calculated according to the Cox regression analyses. A nomogram model was conducted by R 3.6.0 software [21]. *p* < 0.05 was considered to be statistically significant.

## RESULTS

### ROC analysis and AUC comparison

**FIGURE 1:**
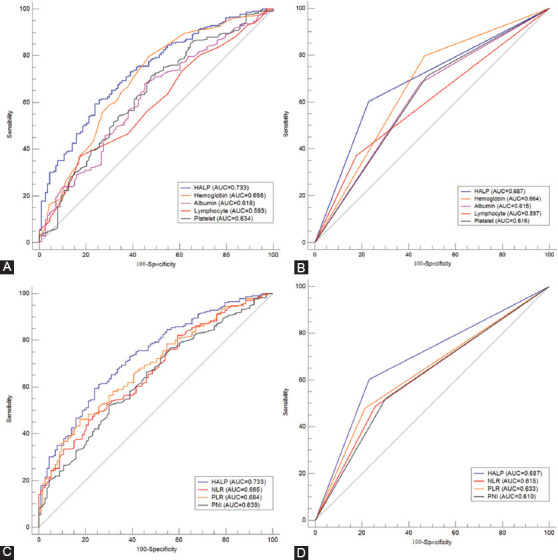
The receiver operating characteristic (ROC) curves of hemoglobin, albumin, lymphocyte and platelet (HALP), and hemoglobin, albumin, lymphocyte and platelet (A for continuous and B for categorical). The ROC curves of HALP and neutrophil to lymphocyte ratio, platelet to lymphocyte ratio and prognostic nutritional index (C for continuous and D for categorical).

The ROC curves regarding postoperative HALP and its components, including albumin, lymphocyte, hemoglobin, and platelet, were shown in Figure 1A-B (A for continuous and B for categorical). Compared with hemoglobin (continuous AUC =0.698; categorical AUC =0.664), albumin (continuous AUC =0.618; categorical AUC =0.615), lymphocyte (continuous AUC =0.593; categorical AUC =0.597), and platelet (continuous AUC =0.634; categorical AUC =0.616), HALP had the largest AUC (0.733 for continuous and 0.687 for categorical) according to the ROC curves. The comparisons between HALP and other three different prognostic scores (NLR, PLR, and PNI) were also analyzed. The results in Figure 1C-D clearly showed that HALP was superior to other prognostic scores (C for continuous and D for categorical). The AUC comparisons between HALP and other variables were shown in Table 1.

**TABLE 1:**
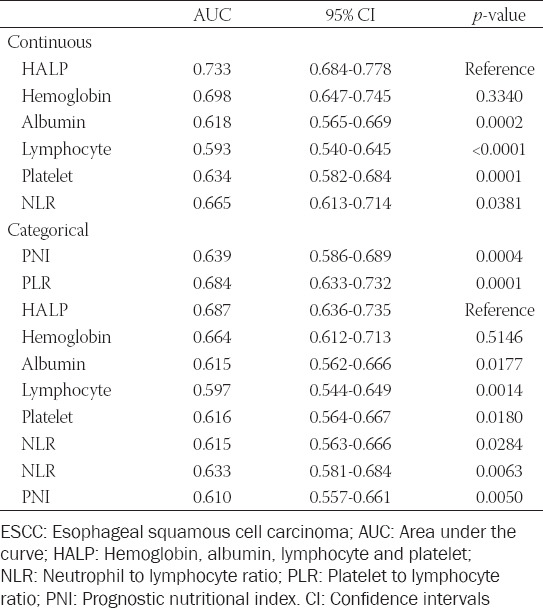
Comparison of AUC areas between HALP and other markers in ESCC

### Patient characteristics

**FIGURE 2:**
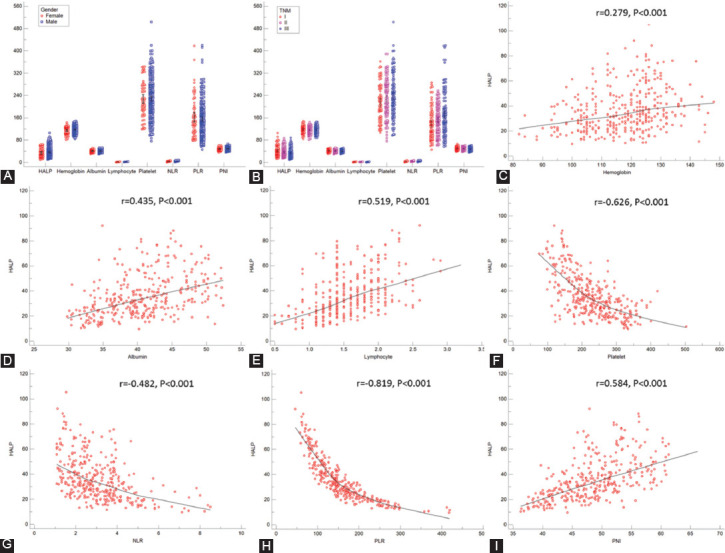
The distribution diagrams of hemoglobin, albumin, lymphocyte and platelet (HALP) as well as its components, neutrophil to lymphocyte ratio (NLR), platelet to lymphocyte ratio (PLR), and prognostic nutritional index (PNI) grouped by gender (A) and TNM stage (B). The correlation diagrams between HALP and hemoglobin (C), albumin (D), lymphocyte (E), platelet (F), NLR (G), PLR (H), and PNI (I).

The median age of enrolled patients in the current study was 59 years (range: 36-80 years), and the median time of follow-up was 34 months (range: 4-94 months). In the current study, there were 297 males (83.7%) and 58 females (16.3%). The mean values of HALP were 117.1 ± 12.3 g/L (median: 119 g/L), 40.7 ± 5.4 g/L (median: 40.6 g/L), 1.56 ± 0.43 10^^9^/L (median: 1.50 10^^9^/L), and 228.2 ± 71.0 10^^9^/L (median: 224 10^^9^/L), respectively. The mean value of HALP was 36.1 ± 17.1 (median: 32.4; range: 9.6-105.4). The distribution diagrams of HALP, as well as its components of lymphocyte, albumin, hemoglobin, and platelet, NLR, PLR, and PNI grouped by gender and TNM, were shown in Figure 2A-B. There was a significant correlation between hemoglobin and gender was observed (114.2 ± 13.7 g/L for female and 117.8 ± 11.9 g/L for male, *p* = 0.033). However, no significant correlations were found in HALP and other variables grouped by gender. On the contrary, no significant correlations were found in hemoglobin based on TNM stage.

The correlation diagrams between HALP and HALP, NLR, PLR, and PNI were shown in Figure 2C-I. The results in the current study revealed that HALP was negatively correlated with platelet (r = −0.626, *p* < 0.001), NLR (r = −0.482, *p* < 0.001), and PLR (r = −0.819, *p* < 0.001), respectively. Positive correlations were found between HALP and hemoglobin (r = 0.279, *p* < 0.001), albumin (r = 0.435, *p* < 0.001), lymphocyte (r = 0.519, *p* < 0.001), and PNI (r = 0.584, *p* < 0.001), respectively.

The optimal cut-off points according to the ROC curves in the current study for HALP, NLR, PLR, and PNI were 31.8, 3.2, 162.5, and 47.6, respectively. Then patients assigned to HALP using the cut-off value of 31.8 and categorized into the low and high groups (low group: HALP ≤31.8 and high group HALP >31.8). The comparisons between patient characteristics grouped by HALP were shown in Table 2.

**TABLE 2:**
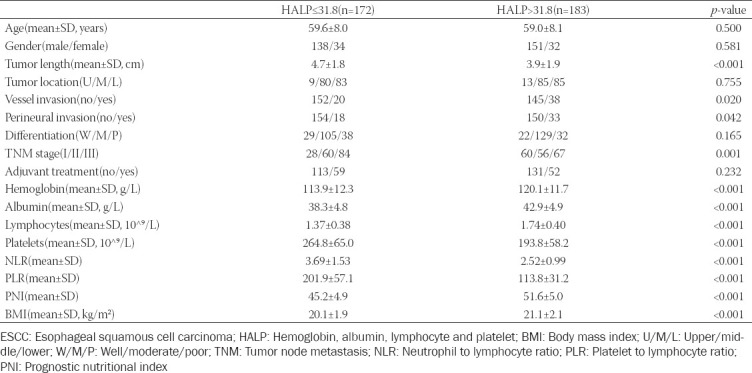
Clinical characteristics in patients with ESCC according to HALP score

### CSS analysis and subgroup analysis

**FIGURE 3:**
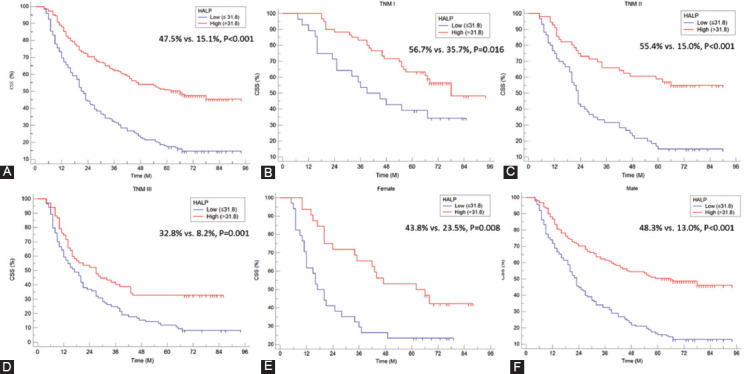
The cancer-specific survival grouped by hemoglobin, albumin, lymphocyte and platelet according to the Kaplan–Meier analysis (A). Subgroup analyses based on TNM stage (TNM I: B; TNM II: C; TNM III: D) and gender (female: E; male: F) regarding the cancer-specific survival grouped by hemoglobin, albumin, lymphocyte and platelet.

In the current study, the 5-year CSS was 31.8%. The 5-year CSS for postoperative HALP low and high group were 15.1% and 47.5%, respectively (*p* < 0.001, Figure 3A). To investigate the effect of postoperative HALP on prognosis in different tumor stage cohorts, we performed the subgroup analysis based on stratification of the total cohort according to different tumor stages (TNM I-III). The results also demonstrated that postoperative HALP was significantly related to CSS in subgroup analyses according to TNM stage in resectable patients with ESCC (TNM I: *p* = 0.016; TNM II: *p* < 0.001; TNM III: *p* = 0.001) (Figure 3B-D). Moreover, another subgroup analysis based on gender was also performed in Figure 3E-F. The results also demonstrated that postoperative HALP was significantly associated to CSS (female: *p* = 0.008; male: *p* < 0.001). These findings suggested that HALP had reliable abilities to predict prognosis in resected ESCC in any stage or gender.

### Univariate and multivariate analysis for CSS

To investigate the significance of postoperative HALP and other prognostic scores regarding CSS in this study, univariate and multivariate cox regression analyses were performed. According to the univariate analysis, it is recognized that postoperative HALP score (HR = 2.458, 95% CI: 1.895-3.189, *p* < 0.001), as well as NLR (HR =1.793, 95% CI: 1.393-2.310, *p* < 0.001), PLR (HR =2.055, 95% CI: 1.594-2.649, *p* < 0.001), and PNI (HR =1.588, 95% CI: 1.233-2.046, *p* < 0.001) were significantly associated with CSS in resected ESCC patients (Table 3). Multivariate analysis confirmed that postoperative HALP score (HR =2.023, 95% CI: 1.549-2.644, *p* < 0.001), but not for NLR, PLR, or PNI, was an independent prognostic marker in resectable ESCC patients regarding CSS (Table 4).

**TABLE 3:**
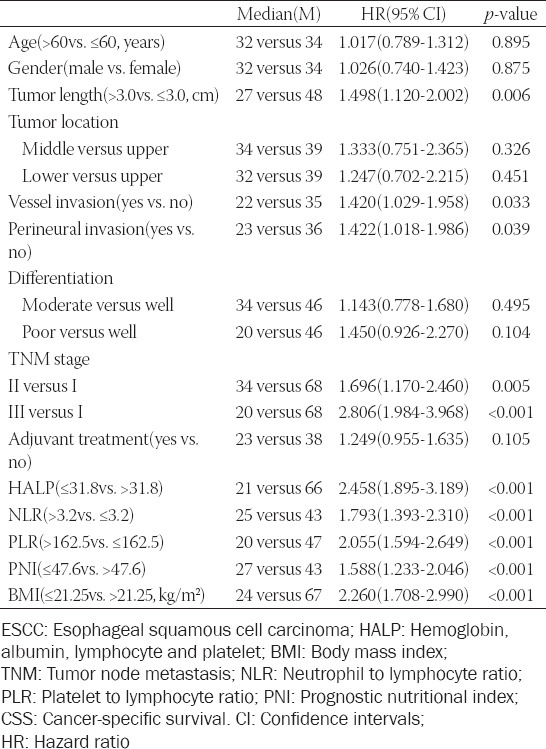
Univariate analyses of CSS in ESCC patients

**TABLE 4:**
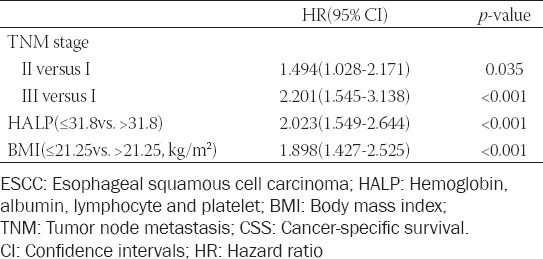
Multivariate analyses of CSS in ESCC patients

### Nomogram analysis

**FIGURE 4:**
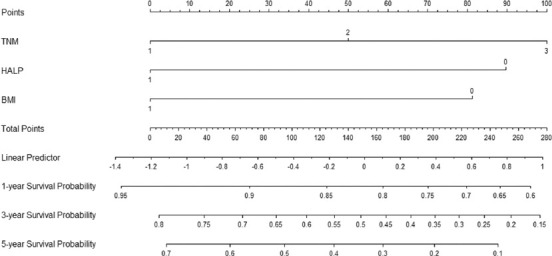
A prognostic nomogram model including hemoglobin, albumin, lymphocyte and platelet, TNM, and BMI was conducted to predict the risk of death probability for patients with resected esophageal squamous cell carcinoma.

To better evaluate the prognostic values and clinical significance of postoperative HALP score and other independent prognostic factors, a prognostic nomogram model was developed for patients with resectable ESCC in the current study. The significant independent prognostic factors in multivariate analyses (HALP, body mass index [BMI], and TNM stage) were included in the current nomogram model to perform individualized survival prediction for patients with resectable ESCC (Figure 4).

## DISCUSSION

The present study explored the association between postoperative HALP and clinical characteristics as well as prognosis in resectable ESCC patients. The results demonstrated that postoperative HALP was still a useful independent prognostic score. Compared with its components, including lymphocyte, albumin, hemoglobin, and platelet in both continuous and categorical status, HALP had the largest AUC (0.733 for continuous and 0.687 for categorical) according to the ROC curves. Moreover, compared with the other three different prognostic scores (NLR, PLR, and PNI), the results clearly showed that postoperative HALP was superior to other prognostic scores. Overall, the results in this study revealed that postoperative HALP has higher prediction accuracy based on ROC curves than other related markers in CSS.

The HALP, consisting of lymphocyte, albumin, hemoglobin, and platelet, was recently used in patients with several cancers [13-17]. A study including 1360 patients in resected renal cell carcinoma revealed that the postoperative HALP score was closely associated with clinical characteristics and was an independent significant predictor of CSS [22]. The HALP was also confirmed in another study, including 516 patients with bladder cancer who received radical cystectomy [23]. Moreover, the results also revealed that the postoperative HALP score may be a useful prognostic score regarding overall survival in patients with bladder cancer with radical resection [23]. In daily clinical practice, it is well known that lymphocyte, albumin, hemoglobin, and platelet are common clinical biomarkers. To the best of our knowledge, only one study, including 39 inoperable patients with ESCC, investigated the clinical significance and prognosis of HALP in predicting platinum-based chemoradiotherapy response [17]. They demonstrated that HALP was associated with progression-free survival in male patients but not associated with overall survival. The previous study of ESCC focused on inoperable patients with a small sample (39 patients). We hypothesized that HALP score could achieve the same significant prognostic value in patients with resectable ESCC. Therefore, we performed the current study to explore the relationship between CSS and clinical characteristics, as well as postoperative HALP, in resectable ESCC patients.

In our study, the 5-year CSS for postoperative HALP > 31.8 (47.5%) was higher than that for postoperative HALP ≤ 31.8 (15.1%). The same results were also found in other studies in patients with metastatic prostate cancer (cut-off value of 32.4), pancreatic cancer (cut-off value of 44.56), small cell lung cancer (cut-off value of 25.8), and bladder cancer (cut-off value of 22.2) [13-15,23]. Previous published studies have revealed that low levels of lymphocyte, albumin, and hemoglobin were associated with poor survival in various cancers [24-26]. HALP was a composite index regarding the above indexes with a stronger prognostic ability. Subgroup analysis based on stage and gender suggested that postoperative HALP had reliable abilities to predict prognosis in resected ESCC patients in any stage or gender. Multivariate analyses confirmed that postoperative HALP was an independent prognostic marker regarding CSS.

It is widely accepted that inflammation and nutrition are associated with tumor prognosis. Recently, published researches demonstrated that a range of inflammation-related and/or nutrition-related indicators, such as PNI, PLR, and NLR, is related to tumor prognosis in various cancers, as well as ESCC [8-12]. Compared with other prognostic scores, HALP was considered to be the highest-scoring system for grouping patients with the same prognosis. A study including 82 patients with metastatic prostate cancer revealed that the HALP score had a higher predictive ability than those of NLR and PLR [13]. The same results were also confirmed in another study, including 582 patients with radical resection in pancreatic cancer [14]. Recently, the lung immune prognostic index (LIPI), consisting of lactate dehydrogenase and derived NLR (dNLR), was firstly reported in patients treated with an immune checkpoint inhibitor in metastatic non-small cell lung cancer [27]. LIPI was not a unique prognostic indicator for lung cancer. HALP was a biomarker with nutritional and inflammatory status, while LIPI was considered as a novel immunotherapy biomarker. We recently reported that LIPI was still a potential independent prognostic marker in patients with resected ESCC [28]. However, the correlations between HALP and HALP need to be expected with more cohort studies. The AUCs for postoperative HALP and its components of lymphocyte, platelet, hemoglobin, and albumin, as well as NLR, PLR, and PNI were calculated and compared in both continuous and categorical statuses in the current study. The results demonstrated that postoperative HALP had the largest AUC (0.733 for continuous and 0.687 for categorical) according to the ROC curves. Moreover, compared with the other three different prognostic scores (NLR, PLR, and PNI), the results clearly revealed that the postoperative HALP was superior to other prognostic scores.

Cancer prognosis is determined not only by pathological stage but also by host factors, especially for nutritional and/or inflammatory status [29,30]. HALP score, including albumin, lymphocyte, hemoglobin, and platelet, reflects both nutritional and inflammatory status. However, the mechanism of HALP in cancer prognosis has not been thoroughly studied. Low hemoglobin is present in many types of cancers. It has been proposed that tumor necrosis factor-a and interleukin-6 (IL-6), secreted by tumor cells, can reduce hemoglobin levels by altering the hematopoietic environment [31]. The serum albumin is known as a reliable indicator of nutritional and inflammatory status. It has been noted that inflammatory cells, recruited by tumor cells, can suppress the action of cytotoxic lymphocytes. Lymphocytes can cause systemic inflammation by releasing transforming growth factor-b and IL-10 [32]. Platelet activation leads to the release of angiogenic growth factors to increase vascular permeability [33].

A previous study reported that there was a significant correlation between sex and HALP [14]. The results revealed that male patients had a higher level of HALP than female patients. They concluded that the significant association between sex and HALP was mainly due to the differences in hemoglobin between male and female (female: 127.1 g/L and male: 138.1 g/L, *p* < 0.001). However, no significant correlations were found between HALP and sex in other studies [13,15-17] as well as the current study, but a significant association between sex and hemoglobin was found in the current study (114.2 ± 13.7 g/L for female and 117.8 ± 11.9 g/L for male, *p* = 0.033). The mean value of hemoglobin in the current study was lower than that in the previous study [14], mainly because most patients in ESCC had malnutrition.

There is no general consensus on the influence of BMI regarding EC prognosis. Several studies revealed that patients with lower BMI had a significantly poor prognosis than those with higher BMI [34,35]. The same results were found in the current study (cut-off value of 21.25 kg/m^2^). However, other studies revealed conflicting results. A study including 736 patients with EC divided into three groups according to BMI: Normal weight (<25 kg/m^2^), overweight (25-30 kg/m^2^) or obese (≥30 kg/m^2^). The results demonstrated that 5-year survival rates were not influenced by the postoperative BMI [36]. Another study, including 556 EC patients with radical resection, also reported that BMI (also divided into three groups) was not of prognostic value with regard to prognosis in patients with radical resection [37]. A clinical-based cohort including 2031 consecutive EC patients and meta-analysis including 14 studies revealed that postoperative BMI, divided into three groups according to Asian-specific cut-off values: <18.5 kg/m^2^, 18.5-23 kg/m^2^, or ≥23 kg/m^2^, was an independent prognostic indicator [38]. The main reason regarding the controversial results was that different cut-off values (the mean BMI was relatively low in China) of BMI in different studies.

Previous published study reported that postoperative HALP may have important clinical implications. They believed that improvement of inflammation and malnutrition can improve patient prognosis and prevent post-operative complications. It is worth noting that the results of the present study regarding postoperative HALP score have potential applications in the clinical practice in resectable ESCC patients. Postoperative identification of nutritional and inflammatory status could have several advantages in clinical practice, including prognosis and treatment. At present, we are investigating postoperative HALP score in our patients with ESCC to preoperatively improve nutritional and inflammatory status in patients who have a low score of postoperative HALP (≤31.8) before surgery. If patients with a low score of postoperative HALP (≤31.8), it is recommended to use anti-inflammatory drugs or other non-steroidal drugs to alleviate systemic inflammation and to raise the levels of hemoglobin and albumin to improve the status of malnutrition before surgery, or to conduct adjuvant therapy after surgery.

Some limitations should be noted in the current study. First, this study is a single-center study in a small sample with a retrospective character. Second, the levels of HALP may be influenced by various statuses, which will limit the application of postoperative HALP. Third, patients treated with postoperative therapy were excluded in the current study, which may have influenced results. Finally, the validity of postoperative HALP score needs to be expected with more cohort studies.

## CONCLUSION

The present study concluded the association between postoperative HALP and prognosis in resected ESCC. The results confirmed that postoperative HALP, a simple and easily obtained prognostic score, is still an independent prognostic score in patients with resectable ESCC. The validity of postoperative HALP score needs to be expected with more cohort studies.
